# A Clinical Prediction Model for Early Colectomy in Patients With Severe Ulcerative Colitis Treated With Tacrolimus

**DOI:** 10.1002/jgh3.70297

**Published:** 2025-10-24

**Authors:** Hikaru Shimizu, Kazuki Kakimoto, Mai Utsumi, Suzune Sugishima, Hideaki Mitooka, Noboru Mizuta, Ryosuke Yamaguchi, Masatoshi Kaizuka, Koji Nishida, Keijiro Numa, Naohiko Kinoshita, Kei Nakazawa, Ryoji Koshiba, Yuki Hirata, Takako Miyazaki, Shiro Nakamura, Hiroki Nishikawa

**Affiliations:** ^1^ 2nd Department of Internal Medicine Osaka Medical and Pharmaceutical University Takatsuki Japan

**Keywords:** colectomy, prediction model, tacrolimus, ulcerative colitis

## Abstract

**Background/Aims:**

Tacrolimus is an effective treatment option for refractory ulcerative colitis; however, some patients still require colectomy due to insufficient response. Early assessment of surgical risk is clinically important, as delayed decision‐making may worsen the patient's condition and increase the risk of postoperative complications. This study aimed to identify predictors of colectomy within 3 months of initiating tacrolimus therapy and to develop a clinically applicable prediction model.

**Methods:**

We conducted a retrospective analysis of hospitalized patients with severe ulcerative colitis treated with tacrolimus between 2011 and 2025. Fourteen clinical background variables were evaluated using LASSO‐penalized logistic regression with cross‐validation to construct the prediction model.

**Results:**

Among 114 patients, 24 (21.1%) underwent colectomy, including 16 (14.0%) within 3 months of treatment initiation. The LASSO regression identified three predictive variables: serum albumin level, hemoglobin level, and age at tacrolimus initiation. The resulting model demonstrated good discriminative performance, with an area under the curve of 0.78. Using a cutoff value of logit(*p*), the model achieved a sensitivity of 87.5% and a specificity of 63.4%. Kaplan–Meier analysis revealed a significantly higher cumulative colectomy rate in the high‐risk group (*p* < 0.001), supporting the model's predictive utility.

**Conclusion:**

We developed a clinical prediction model that accurately estimates the risk of early colectomy based on baseline clinical factors at the start of tacrolimus therapy. This model may serve as a practical tool to guide decision‐making regarding surgical timing and overall treatment strategy.

## 1 Introduction

Ulcerative colitis (UC) is a chronic inflammatory disorder of the colon characterized by an unpredictable course of relapse and remission, with an unknown etiology. In recent years, the introduction of biologics and Janus kinase (JAK) inhibitors has significantly expanded the treatment options for UC. However, a subset of patients remains refractory to pharmacologic therapy and ultimately requires surgical intervention, most commonly total colectomy [1, 2].

In Japan, tacrolimus is frequently used in patients with severe UC, particularly in cases of steroid‐resistant or steroid‐dependent disease [3, 4]. Like cyclosporine A (CsA), tacrolimus is a calcineurin inhibitor that modulates immune responses and suppresses inflammation by inhibiting interleukin‐2 production and T‐cell activation [5, 6, 7]. A randomized, double‐blind, controlled trial by Ogata et al. demonstrated the efficacy of tacrolimus in inducing remission in patients with steroid‐refractory UC [8]. Nevertheless, some patients do not respond to tacrolimus and eventually require surgery [9]. Delayed evaluation of treatment response or surgical indication may lead to deterioration of the patient's general condition, progression of intestinal damage, and increased risk of postoperative complications. Therefore, early identification of patients at high risk of requiring surgery is essential for optimizing treatment strategies, determining appropriate timing for surgical intervention, and ultimately improving clinical outcomes. This issue remains critically important in real‐world clinical practice.

The ability to predict, at the outset of tacrolimus therapy, whether a patient with UC will ultimately require surgery would be highly valuable from the perspective of personalized medicine. However, only a limited number of studies have addressed this issue to date [10, 11]. In recent years, the importance of personalized medicine supported by predictive modeling has been increasingly recognized, and statistical methods such as machine learning and regularized regression have begun to be applied in medical research [12, 13, 14]. Among these approaches, Least Absolute Shrinkage and Selection Operator (LASSO) has gained attention for its ability to automatically select a subset of predictive variables from a large pool of candidates. This technique enhances predictive accuracy while reducing the risk of overfitting [13]. Applying such an approach to patients receiving tacrolimus therapy may facilitate the identification of baseline predictors of surgical outcomes and support the development of a prediction model at the time of treatment initiation.

In this study, we analyzed a cohort of severe UC patients treated with tacrolimus, using established clinical background variables as candidate predictors. We applied LASSO‐penalized logistic regression with cross‐validation to identify independent predictors of colectomy. Based on these variables, we constructed a prediction model and evaluated its discriminative performance.

## 2 Methods

### 2.1 Study Design

This retrospective study included patients with severe ulcerative colitis who were hospitalized and treated with tacrolimus (TAC) at our institution between March 2011 and March 2025. TAC therapy was initiated at an oral dose of 0.1 mg/kg/day, administered twice daily. Serum trough levels were regularly monitored, and dosages were adjusted accordingly. The target trough concentration was maintained at 10–15 ng/mL for the first 2 weeks, followed by 5–10 ng/mL for the subsequent 3 months. Starting 3 months after initiation, TAC was gradually tapered and subsequently discontinued. Baseline laboratory testing, including markers of systemic inflammation, was performed within 2 days prior to the initiation of TAC therapy.

### 2.2 Outcomes and Definitions

Early colectomy was defined as surgical intervention performed within 3 months of initiating tacrolimus therapy, whereas colectomy referred to surgery performed at any time during the observation period. The primary outcome was to develop a predictive model for early colectomy and to evaluate its discriminative performance using the area under the receiver operating characteristic curve (AUC). Secondary outcomes included the clinical remission rate under tacrolimus treatment; the rates of colectomy within 3 months and over the entire follow‐up period; early colectomy rates stratified by prior treatment history and age; the early colectomy‐free rate according to risk categories defined by the predictive model; and internal validation performance of the model. Clinical remission rates were analyzed using the full analysis set (FAS), which included all patients in whom the efficacy of tacrolimus could be assessed. Clinical disease activity of UC was evaluated using the Mayo score, while endoscopic activity was assessed using the Mayo endoscopic subscore (MES). Severe UC was defined as a Mayo score of 11 or 12. Clinical remission was defined as a partial Mayo (pMayo) score of 2 or less, with each subscore of 1 or less.

### 2.3 Statistical Analysis

Continuous variables were summarized as medians with interquartile ranges and compared between groups using the Mann–Whitney *U* test. Categorical variables were described as frequencies and percentages and analyzed using the chi‐squared or Fisher's exact test, depending on the sample size.

Predictive factors for early colectomy were identified using LASSO‐penalized logistic regression with cross‐validation. For model development, 14 candidate variables were selected based on previously reported risk factors and clinically relevant indicators: age at tacrolimus initiation, age at disease onset, disease duration, disease location, white blood cell count (WBC), hemoglobin, platelet count (PLT), serum albumin, C‐reactive protein (CRP), Mayo score, steroid‐dependent or steroid‐refractory status, concomitant use of systemic steroids, concomitant use of azathioprine, and prior exposure to anti‐TNF‐α antibodies. Logistic regression with L1 regularization and cross‐validation was applied to assess the association between the candidate variables and the likelihood of early colectomy. Log loss was used as the performance metric, and the optimal regularization parameter (*λ*) was determined using five‐fold stratified cross‐validation to minimize log loss. As part of the preprocessing steps, continuous variables were standardized, and categorical variables were encoded using one‐hot encoding. The final LASSO‐penalized logistic regression model was constructed using the selected variables to predict the risk of early colectomy.

Model performance was evaluated by calculating the area under the receiver operating characteristic curve (AUC). The optimal cutoff value for logit(*p*) was determined from the prediction model to maximize classification performance, and the corresponding sensitivity and specificity were calculated. Patients with a predicted logit(*p*) above the cutoff were classified as the high‐risk group, while those with values below the cutoff were classified as the low‐risk group. To evaluate model discrimination and reduce the risk of overfitting, internal validation was performed using five‐fold cross‐validation with the StratifiedKFold method. The AUC across the five folds, along with the corresponding 95% confidence interval, was calculated.

The colectomy‐free rate within 3 months of initiating tacrolimus treatment was estimated using the Kaplan–Meier method, and the two risk groups were compared using the log‐rank test. The index date (start of observation) was defined as the date of tacrolimus initiation, and the event date as the date of colectomy. Patients who did not undergo surgery within 3 months were censored at the earlier of the last follow‐up date or 3 months after treatment initiation. Kaplan–Meier curves were presented with 95% confidence intervals.

Statistical significance was set at *p* < 0.05 (two‐sided). All statistical analyses were conducted using Python (version 3.X) on the Google Colaboratory platform. Commonly used libraries such as pandas, scikit‐learn, statsmodels, matplotlib, and seaborn were utilized. GraphPad Prism 6 was used to generate bar graphs, line graphs, and box‐and‐whisker plots.

## 3 Results

### 3.1 Patient Characteristics and Baseline Laboratory Parameters

Between March 2011 and March 2025, tacrolimus was administered as induction therapy to 157 patients with UC, of whom 114 had severe UC. Baseline demographic and clinical characteristics are summarized in Table 1. The median age was 47 years, and 55.3% of patients were male. The median disease duration was 31 months (interquartile range [IQR]: 8–87), and pancolitis was observed in 78.9% of patients. A total of 66 patients (57.9%) had steroid‐refractory UC, including 17 (14.0%) with steroid‐dependent disease and 49 (40.5%) with steroid‐resistant disease. The median (IQR) C‐reactive protein (CRP) level was 3.4 mg/dL (0.9–10.8), serum albumin was 2.9 g/dL (2.3–3.5), and the Mayo score was 12 [11, 12]. At the time of tacrolimus initiation, 44 patients (36.4%) were receiving systemic corticosteroids, and 21 (17.4%) were on concomitant immunosuppressive therapy. A total of 27 patients (22.3%) had a history of failure with advanced therapies, most of whom (*n* = 24) had previously failed treatment with anti–TNF‐α antibodies.

**TABLE 1:** Demographic and clinical characteristics at baseline.

Number of patients, *n*	114
Male/female, *n*	63/51
Age at tacrolimus initiation, years, median (IQR)	47 (33–63)
Age at onset, years, median (IQR)	40 (27–55)
Duration of disease, months, median (IQR)	31 (8–87)
UC location; pancolitis/left side, *n*	90/24
Steroid dependence/resistance, *n* (%)	66 (57.9)
Steroid dependence, *n* (%)	17 (14.0)
Steroid resistance, *n* (%)	49 (40.5)
WBC (μL), median (IQR)	8915 (6482–11 825)
Neutrophil (μL), median (IQR)	6370 (4375–9331)
Lymphocytes (μL), median (IQR)	1308 (936–1658)
Hb (g/dL), median (IQR)	11.4 (9.9–13.3)
Platelet (10^4^/μL), median (IQR)	36.8 (30.3–46.7)
Albumin (g/dL), median (IQR)	2.9 (2.3–3.5)
CRP (mg/L), median (IQR)	3.4 (0.9–10.8)
Mayo endoscopic subscore, median (IQR)	3 (3–3)
Mayo score, median (IQR)	12 (11–12)
Concomitant 5‐aminosalicylic acid, *n* (%)	92 (76.0)
Concomitant systemic steroids, *n*	44 (36.4)
Concomitant azathioprine, *n*	21 (17.4)
Advanced therapies exposure, *n* (%)	27 (22.3)
Anti‐TNF‐α antibodies exposure, *n* (%)	24 (19.8)
Ustekinumab exposure, *n* (%)	2 (1.7)
Vedolizumab exposure, *n* (%)	4 (3.3)
JAK inhibitor exposure, *n* (%)	10 (8.3)

Abbreviations: CRP, C‐reactive protein; Hb, hemoglobin; IQR, interquartile range; JAK, Janus kinase; TNF, tumor necrosis factor; UC, ulcerative colitis; WBC, white blood cell.

### 3.2 Proportion of Colectomy Following Tacrolimus Treatment

**FIGURE 1:**
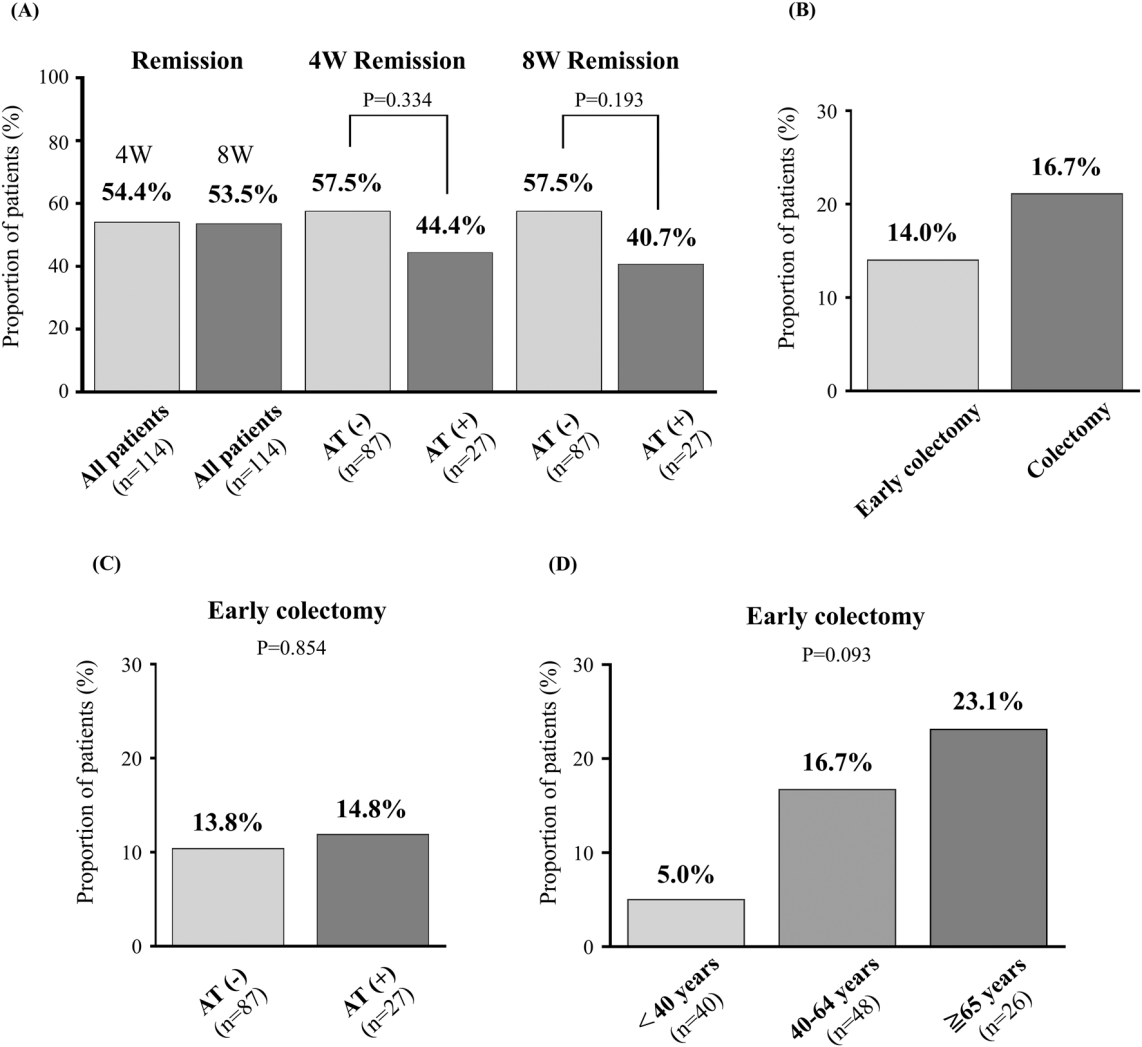
Clinical remission and surgical outcomes after tacrolimus treatment. (A) Clinical remission rate at 8 weeks in the overall population and according to AT exposure status (AT‐exposed vs. AT‐unexposed). (B) Proportion of patients who underwent colectomy during the entire follow‐up period (16.7%) and early colectomy (14.0%), defined as surgery performed within 3 months of tacrolimus initiation. (C) Proportion of patients who underwent early colectomy, stratified by AT exposure status. (D) Proportion of patients who underwent early colectomy, stratified by age group (< 40, 40–64, ≥ 65 years).

The median duration of tacrolimus treatment was 127 days (IQR: 57–165), and the clinical remission rates at 4 and 8 weeks after initiation were 54.4% and 53.5%, respectively (Figure 1A). Although patients with prior exposure to advanced therapies tended to have a lower clinical remission rate at week 8 compared to those without such treatment history (40.7% vs. 57.5%), the difference was not statistically significant (*p* = 0.193) (Figure 1A).

Over the entire observation period (median: 59 months [IQR: 18–107]), 24 patients (21.1%) underwent colectomy, with a median time to surgery of 63 days (IQR: 12–188) (Figure 1B). Of these, 16 patients (14.0%) underwent colectomy within 3 months of starting tacrolimus, with a median time to surgery of 29 days (IQR: 8–62). No association was found between prior exposure to advanced therapies and early colectomy (advanced therapies exposure vs. no exposure: 14.8% vs. 13.8%, *p* = 0.854) (Figure 1C). When patients were stratified by age at the time of tacrolimus initiation, the rates of early colectomy were 5.0% (2/40) in those under 40 years, 16.7% (8/48) in those aged 40–64 years, and 23.1% (6/26) in those aged 65 years or older. Although these differences were not statistically significant, a trend toward a higher rate of early surgery was observed in older patients (*p* = 0.093) (Figure 1F).

### 3.3 Factors Associated With Early Colectomy

At baseline, patients in the early colectomy group had significantly lower hemoglobin and albumin levels compared to those in the non‐early colectomy group. Median hemoglobin levels were 10.1 g/dL (IQR: 9.3–11.1) versus 11.9 g/dL (IQR: 10.2–13.4), *p* = 0.004; median Alb levels were 2.2 g/dL (IQR: 2.0–2.6) versus 3.0 g/dL (IQR: 2.5–3.6), *p* < 0.001 (Table 2). In addition, both age at tacrolimus initiation and age at UC onset were significantly higher in the early colectomy group: 54 years (IQR: 45–67) versus 45 years (IQR: 32–61), *p* = 0.036; and 51 years (IQR: 40–65) versus 37 years (IQR: 25–54), *p* = 0.021.

**TABLE 2:** Baseline characteristics between the early colectomy group and the non‐early colectomy group.

Number of patients, *n* (%)	Early colectomy group (*n* = 16)	Non‐early colectomy group (*n* = 98)	*p*
Male/female, *n*	9/7	54/44	1.0
Age at tacrolimus initiation, years, median (IQR)	54 (45–67)	45 (32–61)	0.036
Age at onset, years, median (IQR)	51 (40–65)	37 (25–54)	0.021
Duration of disease, months, median (IQR)	20 (5–78)	36 (9–89)	0.443
UC location, Pancolitis/Left side, *n*	14/2	76/22	0.516
Steroid dependence/resistance, *n* (%)	10 (62.5)	56 (57.1)	0.789
Steroid dependence, *n* (%)	4 (25.0)	13 (13.3)	
Steroid resistance, *n* (%)	6 (37.5)	43 (43.9)	
WBC (μL), median (IQR)	9635 (8253–10 938)	8635 (6578–11 873)	0.919
Neutrophils (μL), median (IQR)	6929 (4695–8553)	6370 (4467–9467)	0.704
Lymphocytes (μL), median (IQR)	1425 (980–1830)	1279 (911–1622)	0.330
Hb (g/dL), median (IQR)	10.1 (9.3–11.1)	11.9 (10.2–13.4)	0.004
Platelet (10^4^/μL), median (IQR)	38.5 (34.7–49.2)	36.5 (29.9–46.5)	0.633
Albumin (g/dL), median (IQR)	2.2 (2.0–2.6)	3.0 (2.5–3.6)	< 0.001
CRP (mg/L), median (IQR)	6.0 (2.1–12.8)	3.4 (0.8–10.6)	0.205
Mayo endoscopic subscore, median (IQR)	3 (3–3)	3 (3–3)	0.578
Mayo score, median (IQR)	12 (11–12)	12 (11–12)	0.596
Concomitant 5‐aminosalicylic acid, *n* (%)	12 (75.0)	80 (81.6)	0.508
Concomitant systemic steroids, *n* (%)	7 (43.8)	37 (37.8)	0.783
Concomitant azathioprine, *n* (%)	3 (18.8)	18 (18.4)	1.0
Advanced therapies exposure, *n* (%)	4 (25.0)	23 (23.5)	1.0
Anti‐TNF‐α antibodies exposure, *n* (%)	4 (25.0)	20 (20.4)	0.742
Ustekinumab exposure, *n* (%)	0 (0)	2 (2.0)	1.0
Vedolizumab exposure, *n* (%)	1 (6.2)	3 (3.1)	0.459
JAK inhibitor exposure, *n* (%)	1 (6.2)	9 (9.2)	1.0

Abbreviations: CRP, C‐reactive protein; Hb, hemoglobin; IQR, interquartile range; JAK, Janus kinase; TNF, tumor necrosis factor; UC, ulcerative colitis; WBC, white blood cell.

### 3.4 Analysis of Predictive Factors for Early Colectomy Using LASSO Method

**FIGURE 2:**
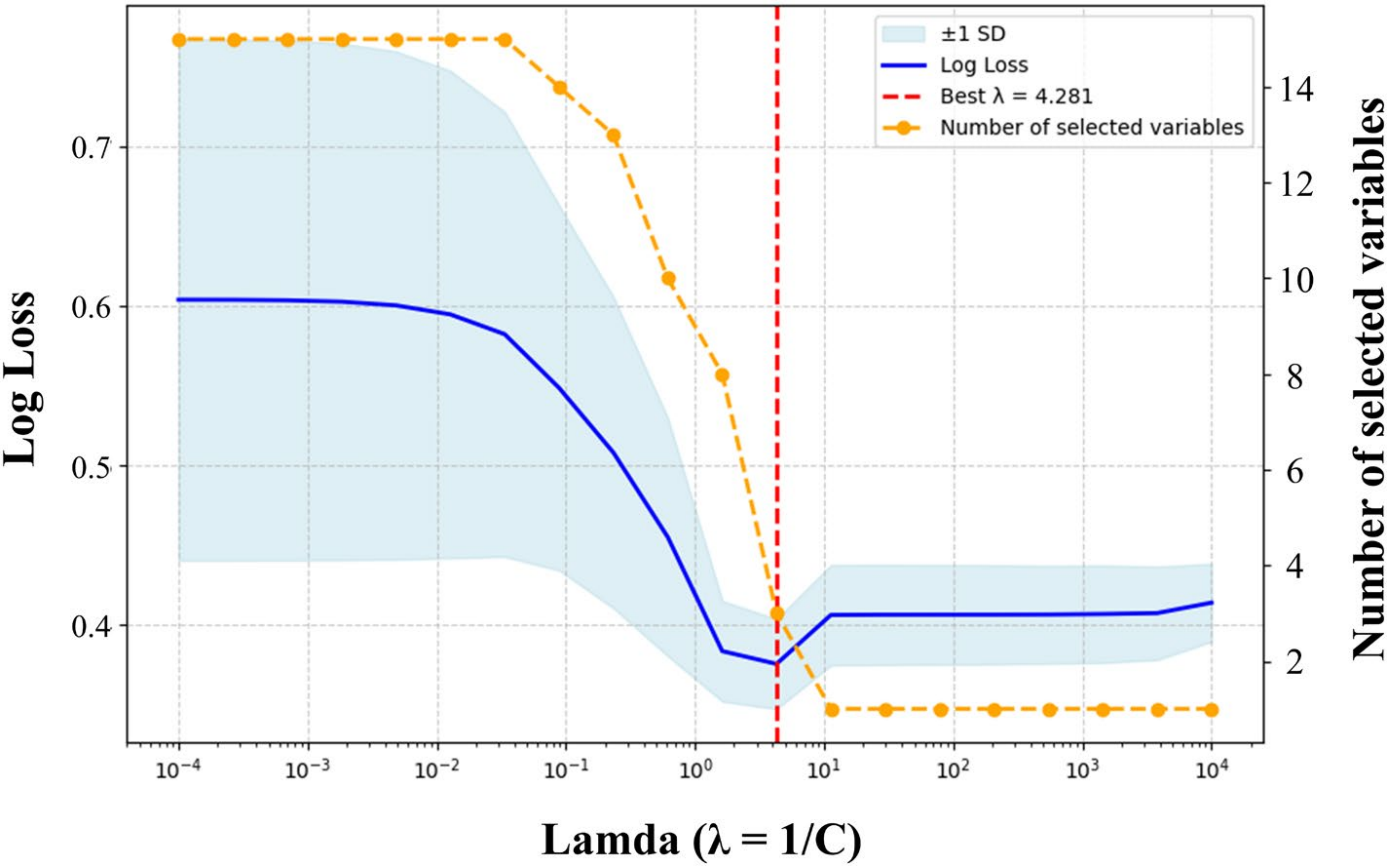
Lambda selection in LASSO‐penalized logistic regression with cross‐validation. Mean log loss and the number of selected variables across a range of regularization parameters (log₁₀(*λ*)), based on 5‐fold cross‐validation. The minimum log loss was observed at *λ* = 4.281, at which point three variables were selected: albumin, hemoglobin, and age.

**FIGURE 3:**
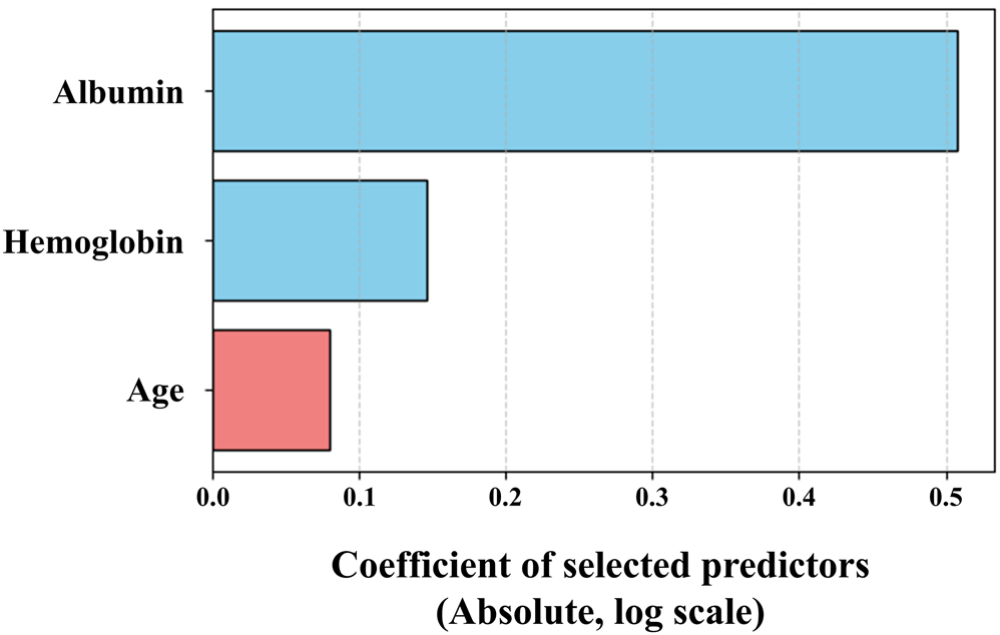
Coefficients of selected predictors in the LASSO‐penalized logistic regression model. Standardized coefficients of the three predictors selected at the optimal lambda value. Albumin and hemoglobin were negatively associated, while age was positively associated with the risk of early colectomy.

LASSO‐penalized logistic regression was performed using 14 candidate predictors to identify factors associated with early colectomy. Figure 2 presents the mean log loss and the number of variables selected at each value of the regularization parameter *λ* (log₁₀(C)), as determined through cross‐validation. The minimum log loss was observed at *λ* = 4.281, indicating optimal model performance under this condition. At this value of *λ*, three variables were selected from the 14 candidates: albumin (Alb), hemoglobin (Hb), and age at tacrolimus initiation (Age). This model was considered to achieve the best balance between bias and variance. The corresponding coefficients for each variable were as follows: Alb, −0.508; Hb, −0.147; and Age, 0.08 (Figure 3). A predictive model for early colectomy was then constructed using these three variables, with the following equation:

$$\text{logit}\left(p\right)=0.686-0.698\times \mathrm{Alb}-0.07\times \mathrm{Hb}+0.004\times \mathrm{Age}$$

**FIGURE 4:**
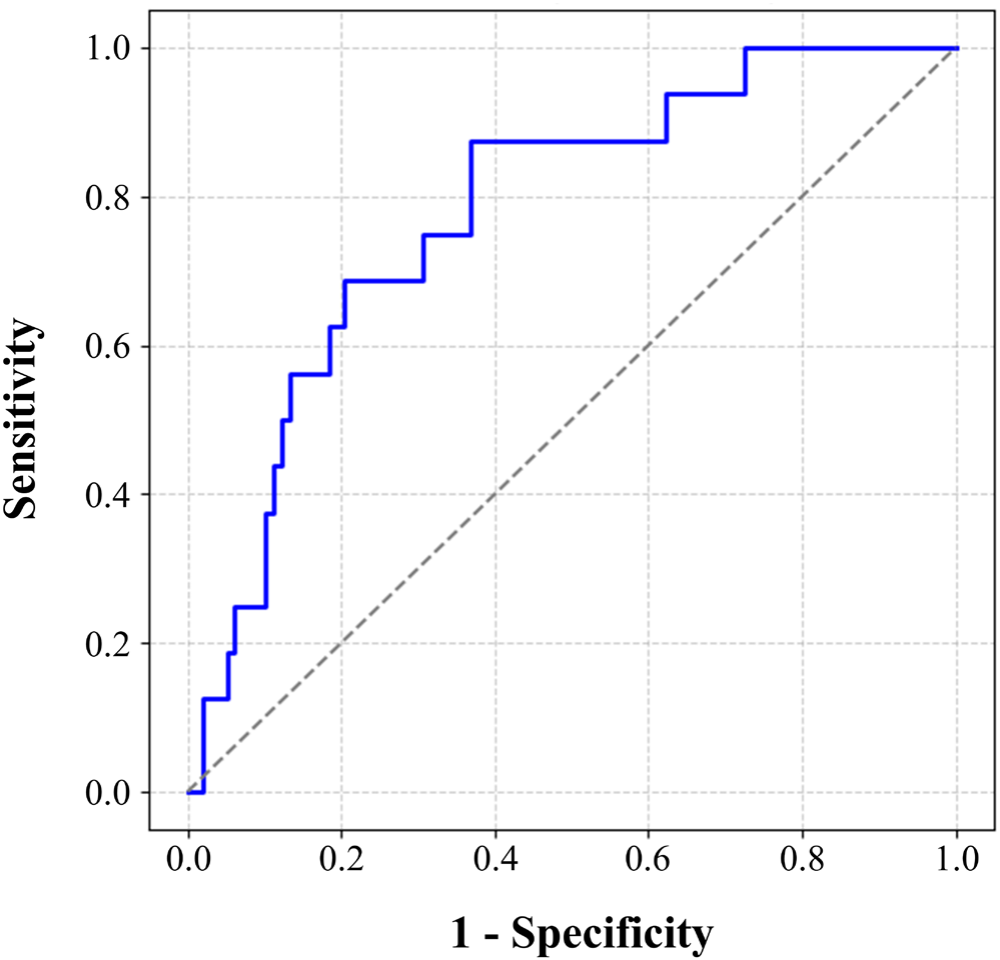
ROC curve of the predictive model for early colectomy. Receiver operating characteristic (ROC) curve of the final logistic regression model. The model demonstrated good discriminative performance, with an AUC of 0.78. The optimal cutoff (logit(*p*) = 0.14) yielded a sensitivity of 87.5% and a specificity of 63.3%.

To evaluate the model's performance, a receiver operating characteristic (ROC) curve was generated. The area under the curve (AUC) was 0.78, indicating good predictive accuracy (Figure 4). When the linear predictor cutoff was set at logit(*p*) = 0.14, the sensitivity was 87.5% and the specificity was 63.3%.

### 3.5 Kaplan–Meier Curve Analysis

**FIGURE 5:**
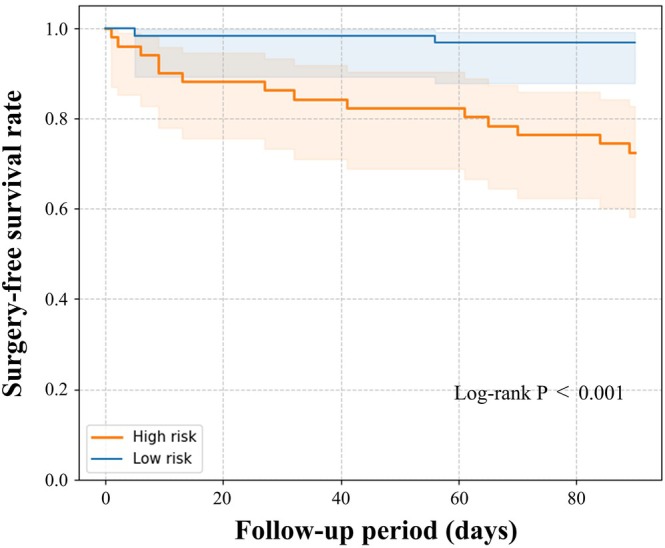
Kaplan–Meier curves for colectomy‐free survival stratified by predicted risk group. Cumulative colectomy‐free survival up to three months after initiation of tacrolimus treatment. Patients classified into the high‐risk group (logit(*p*) ≥ 0.14) had significantly higher colectomy rates compared to those in the low‐risk group (logit(*p*) < 0.14) (*p* < 0.001).

Figure 5 shows the Kaplan–Meier curves for time to colectomy within 3 months after the initiation of tacrolimus therapy. Patients classified into the high‐risk group (logit(*p*) ≥ 0.14) had a significantly higher cumulative incidence of colectomy compared to those in the low‐risk group (logit(*p*) < 0.14) (*p* < 0.001).

### 3.6 Internal Validation

To evaluate the internal validity of the prediction model and assess the risk of overfitting, five‐fold cross‐validation was performed using the StratifiedKFold method. The mean AUC across the folds was 0.73 (95% confidence interval: 0.61–0.86), indicating acceptable discriminative performance. The model demonstrated consistent results across the resampled datasets, suggesting a low risk of overfitting.

## 4 Discussion

In this study, we investigated risk factors associated with early colectomy in patients with moderate to severe ulcerative colitis treated with tacrolimus and developed a predictive model based on these factors. Using LASSO‐penalized logistic regression with cross‐validation, three variables were selected: serum albumin, hemoglobin, and age at tacrolimus initiation. The magnitude of each coefficient indicated the strength of its association with the risk of early colectomy. The prediction model incorporating these three factors demonstrated good discriminative performance, with an area under the receiver operating characteristic curve (AUC) of 0.78. Kaplan–Meier analysis based on the model's risk stratification showed that patients in the high‐risk group had a significantly higher cumulative incidence of colectomy within 3 months after starting tacrolimus, while those in the low‐risk group had a higher colectomy‐free rate. These findings suggest that the model may serve as a clinically useful tool for stratifying patients according to their risk of colectomy. Such stratification could aid in timely decision‐making regarding treatment strategies and the optimal timing of surgical intervention.

While tacrolimus has demonstrated efficacy in patients with refractory or severe ulcerative colitis, a substantial proportion of cases ultimately require surgical intervention due to an insufficient treatment response [9, 10, 11]. Therefore, early identification of high‐risk cases in which surgery is likely to be unavoidable from the outset of treatment may help guide appropriate treatment selection, optimize surgical timing, and ultimately improve patient outcomes. Tacrolimus is a fast‐acting calcineurin inhibitor, and many patients are known to show an initial response within 2 weeks of initiation [10, 15]. The decision regarding whether remission induction has been achieved is typically made within 4–8 weeks after starting treatment [16, 17, 18]. If adequate efficacy is not observed beyond this period, the disease is considered treatment‐refractory. In such cases, switching to an alternative therapy or considering surgical intervention becomes necessary. Ban et al. defined early colectomy as surgery performed within 60 days of initiating tacrolimus [11]. Similarly, in studies investigating anti‐TNF agents and other therapies, colectomy within 90 days of initiation has been used to define early surgery [19]. Based on these reports, the present study defined early colectomy as colectomy performed within 3 months after the initiation of tacrolimus treatment.

Several previous studies have examined factors associated with colectomy in patients with UC treated with calcineurin inhibitors [10, 11]. In the present study, we selected 14 baseline clinical variables considered potentially related to colectomy at the initiation of tacrolimus treatment and constructed a predictive model using LASSO regression. Compared with conventional logistic regression, the LASSO method offers several advantages: it ensures objectivity through automated variable selection using machine learning, allows optimal variable selection by accounting for inter‐variable correlations, and reduces the risk of overfitting [13]. Based on the three variables ultimately identified, we developed a scoring system applicable in clinical settings. This model enables straightforward stratification of high‐risk patients and provides a simple yet practical tool for predicting the need for surgery.

In UC, several studies have shown that low serum albumin and hemoglobin levels indicate greater disease severity and are associated with an increased likelihood of requiring surgery, regardless of the treatment modality, including tacrolimus [20, 21, 22]. A meta‐analysis by Zheng et al. demonstrated a significant association between low albumin levels and a higher risk of colectomy (OR 0.39, 95% CI: 0.26–0.59), identifying albumin < 3.0 g/dL as an independent predictive factor [21]. In patients with highly active UC, hemoglobin levels are often decreased due to impaired iron utilization caused by chronic inflammation or gastrointestinal bleeding, and low hemoglobin has been reported as a potential biomarker reflecting treatment resistance [21, 23]. Since this study included only patients with severe UC, all cases had high Mayo scores of 11 or 12. Consequently, the Mayo score itself was likely excluded as a predictive factor for colectomy in the LASSO regression analysis. Instead, serum albumin and hemoglobin levels—both closely linked to disease activity—were selected as predictors. These findings are consistent with previous reports, further suggesting that low serum albumin and hemoglobin levels may serve as important predictors of colectomy in patients with severe UC treated with tacrolimus.

It is noteworthy that age was selected as a predictor of early colectomy in our analysis. Although the stratified analysis by age group did not show a statistically significant difference in overall colectomy rates, the incidence of early colectomy was numerically higher in older patients. Older adults are known to exhibit reduced responsiveness to corticosteroids and biologics, potentially due to age‐related changes in immune function and the presence of comorbidities [24, 25, 26]. These factors may increase the likelihood of requiring urgent surgical intervention. In addition, treatment options for elderly patients are often more limited, and colectomy may be considered earlier due to concerns about infections or drug‐related adverse events. Our findings suggest that, particularly in elderly patients with UC receiving tacrolimus, careful assessment of overall clinical status and early evaluation of treatment response are essential for optimal management and timely clinical decision‐making.

In a multicenter study conducted in 2021, Oshima et al. reported that patients with ulcerative colitis who had prior exposure to anti–TNF‐α antibodies had significantly lower clinical remission rates at 12 weeks following the initiation of tacrolimus therapy [27]. In our study, the clinical remission rate at week 8 was numerically lower in patients with prior exposure to advanced therapies. Given that the majority (88.9%) of patients in the advanced‐therapies–exposed group had previously received anti‐TNFα antibodies, our findings are consistent with those of Oshima et al. In contrast, we found no association between prior exposure to advanced therapies and the incidence of early colectomy. These findings suggest that tacrolimus treatment may help avoid short‐term surgery, regardless of a patient's prior exposure to advanced therapies.

The developed prediction model demonstrated clinically useful accuracy, with an AUC of 0.78. Notably, the model was constructed using only three readily obtainable clinical variables, which enhance its feasibility for use in routine clinical practice. In recent years, therapeutic options for UC, including biologics and JAK inhibitors, have expanded rapidly. In the future, the analytical approach employed in this study could be applied to other advanced therapies to enable early prediction of treatment resistance and to optimize therapeutic strategies.

Several limitations of this study should be acknowledged. First, the analysis was retrospective and conducted at a single center, which may have introduced institutional bias related to specific treatment strategies or care protocols. Second, internal validation using stratified five‐fold cross‐validation demonstrated comparable performance (AUC = 0.73), supporting the robustness of the model. However, external validation is warranted to confirm its generalizability. Third, the sample size was relatively small. Further studies involving larger, multicenter datasets are required to validate the broader applicability of this model. Fourth, although the 14 candidate variables used for model development were selected based on existing literature and clinical relevance, other potentially important factors—such as biomarkers or imaging findings—were not included and may also influence surgical decision‐making.

Despite these limitations, this study successfully developed a preoperative prediction model applicable at the time of tacrolimus initiation. The model, based on three readily obtainable clinical variables—albumin, hemoglobin, and age—demonstrated reasonable accuracy in predicting early colectomy. These findings highlight the potential utility of the model in supporting early treatment decision‐making for patients receiving tacrolimus. By identifying high‐risk cases at the outset, the model may help avoid the unnecessary continuation of ineffective therapy and reduce delays in appropriate surgical intervention.

## Ethics Statement

This study was conducted in accordance with the principles outlined in the Declaration of Helsinki. The study protocol was approved by the Ethics Committee of Osaka Medical and Pharmaceutical University (protocol code: 2025‐074). As this was a retrospective study, the requirement for informed consent was waived by the ethics committee, and an opt‐out approach was applied.

## Conflicts of Interest

S.N. reports receiving speaking fees from AbbVie GK, EA Pharma Co. Ltd., Mitsubishi Tanabe Pharma Corporation, Mochida Pharmaceutical Co. Ltd., Takeda Pharmaceutical Co. Ltd., Janssen Pharmaceutical K.K., Kyorin Pharmaceutical Co. Ltd., Gilead Sciences Inc., and Pfizer Inc. The other authors declare no conflicts of interest.

## Data Availability

The data that support the findings of this study are available from the corresponding author upon reasonable request.
